# Enhancement of Synthetic *Trichoderma*-Based Enzyme Mixtures for Biomass Conversion with an Alternative Family 5 Glycosyl Hydrolase from *Sporotrichum thermophile*

**DOI:** 10.1371/journal.pone.0109885

**Published:** 2014-10-08

**Authors:** Zhuoliang Ye, Yun Zheng, Bingyao Li, Melissa S. Borrusch, Reginald Storms, Jonathan D. Walton

**Affiliations:** 1 Department of Energy Great Lakes Bioenergy Research Center and Department of Energy Plant Research Laboratory, Michigan State University, East Lansing, Michigan, United States of America; 2 Centre for Structural and Functional Genomics, Concordia University, Montréal, Quebec, Canada; University of Insubria, Italy

## Abstract

Enzymatic conversion of lignocellulosic materials to fermentable sugars is a limiting step in the production of biofuels from biomass. We show here that combining enzymes from different microbial sources is one way to identify superior enzymes. Extracts of the thermophilic fungus *Sporotrichum thermophile* (synonym *Myceliophthora thermophila*) gave synergistic release of glucose (Glc) and xylose (Xyl) from pretreated corn stover when combined with an 8-component synthetic cocktail of enzymes from *Trichoderma reesei*. The *S. thermophile* extracts were fractionated and an enhancing factor identified as endo-β1,4- glucanase (StCel5A or EG2) of subfamily 5 of Glycosyl Hydrolase family 5 (GH5_5). In multi-component optimization experiments using a standard set of enzymes and either StCel5A or the ortholog from *T. reesei* (TrCel5A), reactions containing StCel5A yielded more Glc and Xyl. In a five-component optimization experiment (i.e., varying four core enzymes and the source of Cel5A), the optimal proportions for TrCel5A vs. StCel5A were similar for Glc yields, but markedly different for Xyl yields. Both enzymes were active on lichenan, glucomannan, and oat β-glucan; however, StCel5A but not TrCel5A was also active on β1,4-mannan, two types of galactomannan, and β1,4-xylan. Phylogenetically, fungal enzymes in GH5_5 sorted into two clades, with StCel5A and TrCel5A belonging to different clades. Structural differences with the potential to account for the differences in performance were deduced based on the known structure of TrCel5A and a homology-based model of StCel5A, including a loop near the active site of TrCel5A and the presence of four additional Trp residues in the active cleft of StCel5A. The results indicate that superior biomass-degrading enzymes can be identified by exploring taxonomic diversity combined with assays in the context of realistic enzyme combinations and realistic substrates. Substrate range may be a key factor contributing to superior performance within GH5_5.

## Introduction

The production of liquid biofuels from lignocellulosic materials such as agricultural and forestry residues requires the conversion of the refractory plant cell wall polysaccharides to fermentable sugars by a combination of thermochemical and enzymatic treatments. Because of the complexity of plant cell walls, multiple enzymes are necessary for efficient conversion, including cellulases such as cellobiohydrolases and endo-β1,4-glucanases, and hemicellulases such as endo-β1,4-xylanases and α-glucuronidases. Different pretreatment and feedstock combinations require enzyme cocktails of different composition and relative proportions [1]–[3].

Currently available cellulase mixtures, most of which are produced by fermentation of filamentous fungi such as *Trichoderma reesei*, contain more than 80 proteins [4]. Beyond the core cellulases and hemicellulases, the contribution of most of these proteins to lignocellulose deconstruction is not well understood. One strategy to improve our understanding of the relative and absolute importance of specific enzymes is to construct synthetic mixtures [5]–[8]. Synthetic mixtures containing up to 16 components have been created and optimized using response surface methodologies and robotic liquid dispensing [5]. Such mixtures present a starting point for the identification and analysis of novel enzymes that enhance biomass conversion as well as for the identification of superior versions of known enzymes. Because of the structural complexity of lignocellulose and the often-observed synergism among different types and sources of cellulases, hemicellulases, and side-chain degrading enzymes [10], [11], it is important to do the screening of novel enzymes in as realistic a context as possible. That is, tests of individual pure enzymes on model substrates do not necessarily give a realistic picture of how enzymes will behave in multi-component mixtures on natural lignocellulosic materials.

In this paper, we report the isolation and identification of an endo-β1,4-glucanase from the fungus *Sporotrichum thermophile* (also known as *Myceliophthora thermophila*, class Sordariomycetes) that gives higher glucose (Glc) yields than the orthologous enzyme from *T. reesei* when tested in synthetic enzyme cocktails on pretreated corn stover. Superiority was correlated with phylogenetic sequence relatedness within subfamily 5 of family 5 of glycosyl hydrolases (GH5_5), substrate range, and several deduced structural features.

## Materials and Methods

### Biological materials

Corn stover was ground to 0.5 mm particle size with a Wiley mill and pretreated with 12.5% alkaline hydrogen peroxide (AHP) as described [12]. The 8-component synthetic enzyme mixture (8-CSM) was assembled from purified enzymes. It contained (by mass) 35% cellobiohydrolase 1 (CBH1), 7% cellobiohydrolase 2 (CBH2; GenBank P07987), 5% endo-β1,4-glucanase 1 (EG1, GenBank AAA34212), 4% β-glucosidase (BG, GenBank AAA18473), 36% AA9 (formerly GH61A, GenBank CAA71999), 2% endo-β1,4-xylanase 2 (EX2, GenBank AAB29346), 9% endo-β1,4-xylanase 3 (EX3; GenBank BAA89465), and 3% β-xylosidase (BX; GenBank CAA93248). CBH1 was obtained from Megazyme, Ltd. (Bray, Ireland) and the other proteins were expressed in *Pichia pastoris*, including *T. reesei* Cel5A (TrCel5A; GenBank ABA64553 or AAA34213), by the method described earlier [13]. Bulk quantities were produced by Lucigen, Inc. (Madison, WI). *S. thermophile* Cel5A (StCel5A) was produced by expression in *A. niger* [14]. BothTrCel5A and StCel5A had their native cellulose binding modules (CBM) and no purification tags.

### Chromatography

AlternaFuel CMAX (lot #MXNA2413C), prepared from *S. thermophile*, was a generous gift of Dyadic International, Inc., Jupiter, FL. Enzyme purification was performed with an Agilent HPLC 1200 system equipped with UV detector and fraction collector. All chromatography columns (Tosoh Bioscience, LLC, King of Prussia, PA.) were 8.0 mm×7.5 cm and packed with TSKgel SuperQ-5PW (anion exchange), TSKgel SP-5PW (cation exchange), or TSKgel Phenyl-5PW (hydrophobic interaction).

Solution A for anion exchange was 25 mM Tris-HCl, pH 8.0, and solution B was the same plus 0.6 M NaCl. Solution A for cation exchange was 25 mM sodium acetate, pH 4.0, and solution B was the same plus 0.6 M NaCl. Solution A for hydrophobic interaction was 0.1 M KH_2_PO_4_, pH 7, plus 1.7 M NH_2_SO_4_, and solution B was water. In all cases, the gradients increased from 0 to 100% solution B in 30 min at a flow rate of 1 mL/min, and then held at 100% solution B another 10 min. The fractions (1 mL) were collected in micro-centrifuge tubes at a rate of 1 mL/min.

### Enzyme activity assays

Each 0.5-ml GENPLAT reaction contained biomass at a final glucan concentration of 2 mg/mL in 20 mM sodium citrate buffer, pH 4.8, containing 10 µg/mL cycloheximide and 10 µg/mL tetracycline. Hydrolysis reactions were performed in 96-deep well plates at 50°C for 24, 48, or 72 hr. At each time point, aliquots were removed and free Glc and xylose (Xyl) measured using enzyme-linked assays as described [5]. Mannose (Man) was measured using the assay kit K-MANGL (Megazyme, Wicklow, Ireland). Every reaction was performed in duplicate, sampled twice, and free Glc, Xyl, or Man measured twice from each sample.

To assay synergistic activity, 8-CSM at a loading of 6.5 µg/mg glucan was mixed with 50 µL of each chromatographic fraction. Controls included each fraction by itself and 8-CSM by itself. Degree of synergy is defined as (Glc released by 8-CSM and fraction together) divided by (Glc released from fraction alone plus 8-CSM alone).

Substrate range was tested using various polysaccharide substrates [15]. Sigmacell Type 20, lichenan (from *Cetraria islandica*), xylan (from birchwood), guar gum (from *Cyamopsis tetragonoloba*), locust bean gum (from *Ceratonia siliqua*), and β1,3-glucan (from *Laminaria digitata*) were obtained from Sigma-Aldrich (St. Louis, MO). β-Glucan (from oat, high viscosity, catalog P-BGOH), xyloglucan (from tamarind, catalog P-XYGLN), β1,4-mannan (galactomannan from *Ceratonia siliqua* treated with β-mannanase and α-galactosidase; catalog P-MANCB) and arabinoxylan (from wheat, catalog P-WAXYH) were obtained from Megazyme. Glucomannan (from roots of *Amorphophallus konjac*) was obtained from a local health food store (NOW Foods, Bloomingdale, IL). Phosphoric-acid swollen cellulose (PASC) was prepared as described [16]. Assay conditions were 5 mg/mL substrate concentration, 5 µg/mL enzyme concentration, pH 5.0, and 50°C in 1.5-mL centrifuge tubes in an incubator rotating at10 rpm. Incubation times were varied from 10 min to 24 hr to insure that all reaction rates were in the linear range. Release of reducing sugars was detected by absorbance at 540 nm after reaction with 3,5-dinitrosalicyclic acid (DNS) [17]. All reducing sugar values are expressed as Glc equivalents. Enzyme reaction rates were analyzed using Microsoft Excel and GraphPad Prism (San Diego, CA).

Lichenan (1 mg/mL) was used as the substrate for the characterization of the pH and temperature optima of StCel5A. For the pH experiment, reactions were carried out at 50°C and pH 3–8, using 50 mM sodium citrate for the pH range 3 to 6, and 50 mM Tris-HCl for pH 7 to 8. For the temperature experiment, reactions were carried out in a total volume of 0.1 mL in 25 mM sodium citrate, pH 4.8. Reducing sugars were quantitated with p-aminohydroxybenzoic acid hydrazide [18].

### Protein analysis

Chromatographic fractions showing synergistic activity were analyzed by SDS-PAGE. After staining with Coomassie Blue R-250, bands were excised, digested with trypsin, and analyzed by mass spectrometry at the MSU Research Technology Support Facility. Results were analyzed with Scaffold (version 4.0.5, Proteome Software Inc., Portland, OR).

Protein concentrations were determined by dye binding with bovine IgG as standard [19]. Protein quantitation was verified with SDS-PAGE and Coomassie staining compared to molecular weight markers of known concentration.

Protein alignments and tree construction were performed using MEGA version 6 [20]. Neighbor-Joining with 500 bootstraps, the Jones-Taylor-Thornton (JTT) substitution model, and partial deletion of gaps (to 284 sites) was used.

### Enzyme optimization for AHP-pretreated corn stover hydrolysis

Optimization of enzyme proportions utilized the high-throughput biomass digestion platform GENPLAT [3], [5]. An augmented quadratic experimental design was determined with Design-Expert software (Stat-Ease Inc., Minneapolis, MN). The hydrolysis was carried out at a substrate concentration of 2 mg/mL glucan and a fixed enzyme loading of 15 mg/gm glucan. Free Glc and Xyl were quantitated with enzyme-linked assays.

### Homology modeling and alignments

Modeling of StCel5A was performed using Swiss-Model [21] with *Thermoascus aurantiacus* Cel5A (TaCel5A; PDB:1GZJ) and TrCel5A (PDB:3QR3) as templates. Structure alignment and superposition of StCel5A and TrCel5A were performed using PyMol version 1.5.0.4 (Schrodinger, LLC; www.schrodinger.com).

## Results

### Separation and identification of an enzyme from *Sporotrichum thermophile* that enhances Glc yields

**Figure 1:**
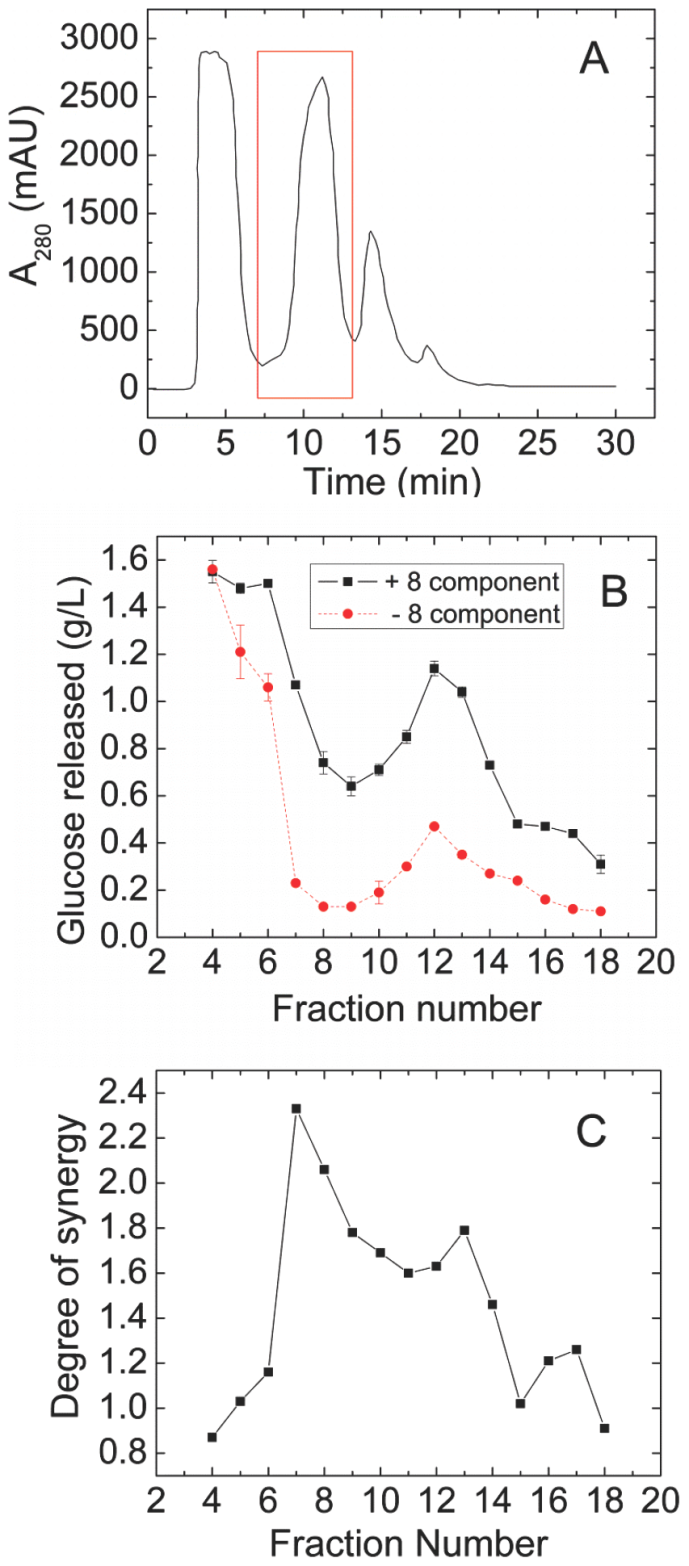
First chromatographic fractionation (anion exchange) of *S. thermophile* secretome. **(A)** UV trace. X-axis is retention time. The red box indicates the fractions collected for second-step separation. **(B)** Glc released from pretreated corn stover after incubation of an aliquot of each fraction from (A) with (black solid line) or without (red dashed line) 8-CSM. Glc released by 8-CSM alone was 0.23±0.01 g/L. Error bars indicate one standard deviation of the mean of triplicates. **(C)** Degree of synergy calculated from (B).

**Figure 2:**
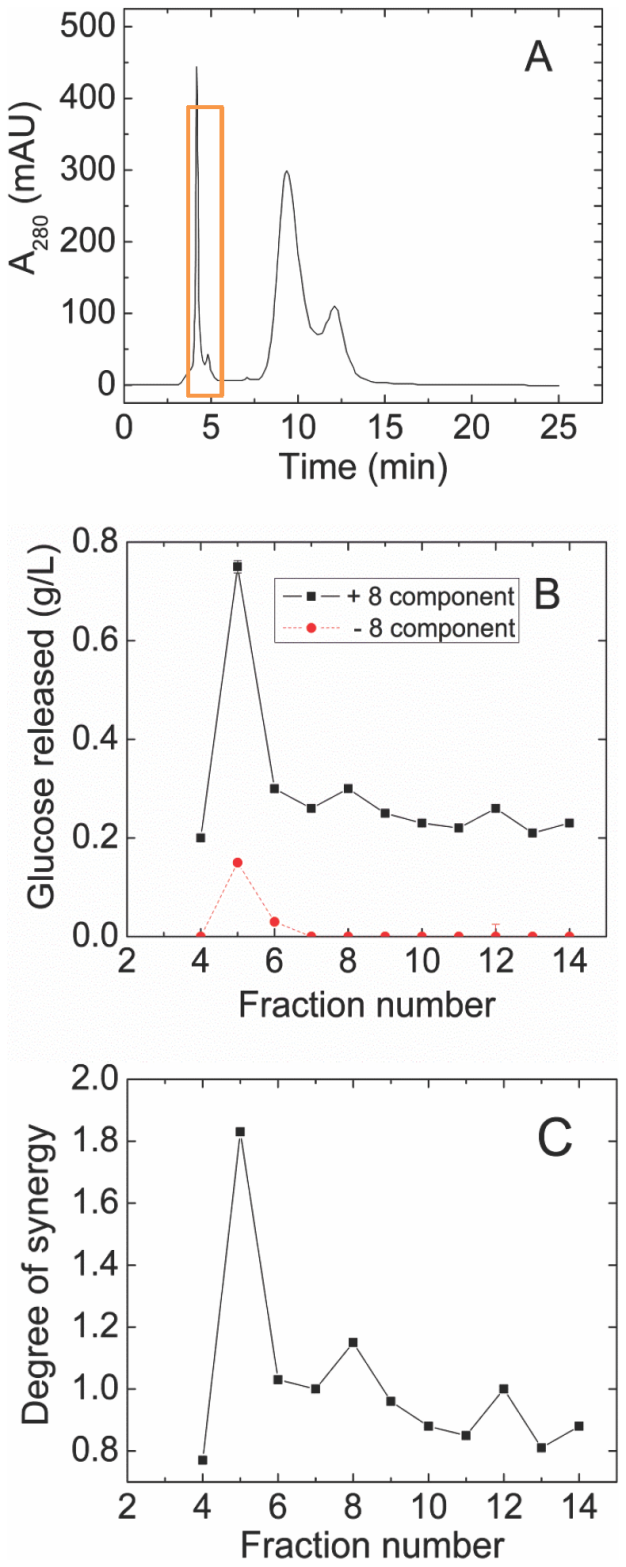
Second fractionation (cation exchange) of fractions 7–10 from the separation shown in Figure 1. **(A)** OD_280_. The red box indicates the fractions collected for the third-step separation. **(B)** Activity with (black solid line) or without (red dashed line) 8-CSM as described in the legend to Fig. 1. Glc released by 8-CSM alone was 0.24±0.01 g/L. **(C)** Degree of synergy calculated from (B).

**Figure 3:**
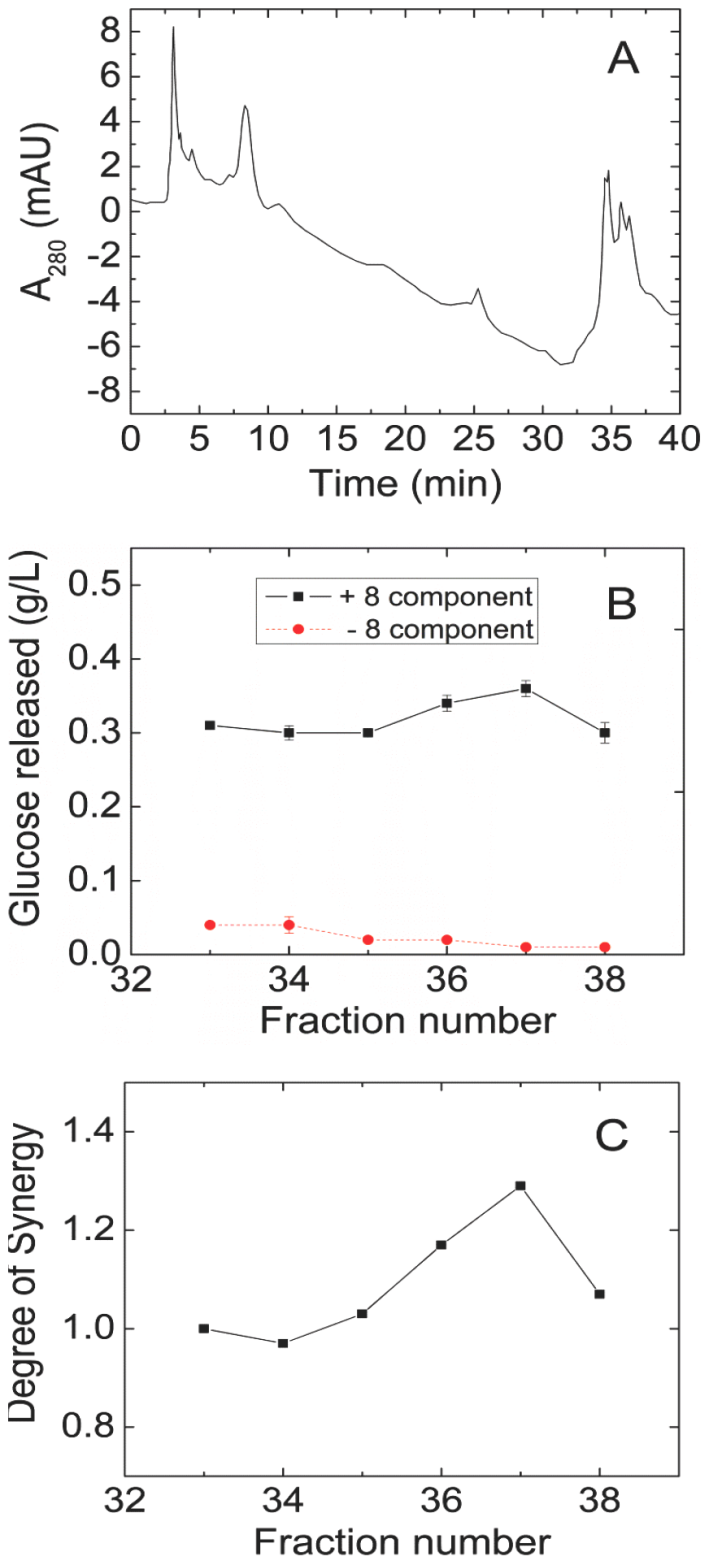
Third fractionation (hydrophobic interaction) of fraction 5 from the separation shown in Figure 2. **(A)** OD_280_. **(B)** Activity with (black solid line) or without (red dashed line) 8-CSM, as described in the legend to Fig. 1. Glc released by 8-CSM alone was 0.26±0.01 g/L. **(C)** Degree of synergy calculated from (B).

A complex mixture of the secretome of *S. thermophile* showed 1.6-fold synergy when added at a 1∶2 ratio with a synthetic enzyme mixture containing CBH1, CBH2, EG1, EX2, EX3, BG, BX, and AA9 from *T. reesei* (see Materials and Methods for enzyme abbreviations and GenBank accession identifiers). The proportions of the different *T. reesei* enzymes in this synthetic mixture, called 8-CSM, were based on earlier studies [5]. The *S. thermophile* extract was fractioned in three chromatographic steps to identify the protein or proteins responsible for the synergism (Figures 1–3). Purification was guided by assaying each chromatographic fraction alone and in combination with the 8-CSM.

After the third separation (Fig. 3), fraction 37 consistently showed the highest synergy. Proteins in this fraction were analyzed by SDS-PAGE and mass-spectrometry proteomics based on the complete genome sequence of *S. thermophile* [22]. In three experiments, an endo-β1,4-glucanase of family 5 (GenBank accession numbers HQ163779, AEO53769, or AEM23898; Joint Genome Institute identifier Spoth2|86753, herein abbreviated StCel5A) was consistently identified as one of the most abundant proteins in the chromatographic fractions showing maximum synergism. For example, in one proteomics experiment, 113 peptides from StCel5A were observed, accounting for 48% of the total peptides with 24% protein coverage. The next most abundant proteins in this experiment were Spoth2|112050 (GH10 xylanase, 53 peptides), Spoth2|66729 (GH6 β-glucanase, 33 peptides), and Spoth|112399 (GH131 β-glucanase, 31 peptides). StCel5A has previously been studied and named EG2 or EG II [14], [23]-[25]. It is endo-acting and shows synergy with CBH1 of *T. reesei* and other β1,4-glucanases on filter paper, Avicel, and pretreated fir wood [14], [24]. Variants of StCel5A with improved specific activity and thermostability have been developed through directed evolution [23].

These results indicated that StCel5A accounts for at least some of the synergism between *S. thermophile* extracts and the synthetic 8-CSM mixture of *T. reesei*, although it does not exclude the existence of other synergistic factors. The best ortholog in *T. reesei* to StCel5A is GenBank accession number ABA64553 (AAA34213), also known as EG2, referred to herein as TrCel5A.

### Enzyme mixture optimization

To further test whether StCel5A was responsible for the observed synergistic effect of the *S. thermophile* secretome, StCel5A was compared side-by-side with TrCel5A at equal molar loadings in mixture optimization experiments [5], [13]. In these experiments, the lower limits of CBH1, EG1 and BG were set to 5% and the lower limit for the other enzymes including StCel5A and Tr Cel5A were set to 0%. Total enzyme loading in every reaction was fixed at 15 mg/g glucan.

**Figure 4:**
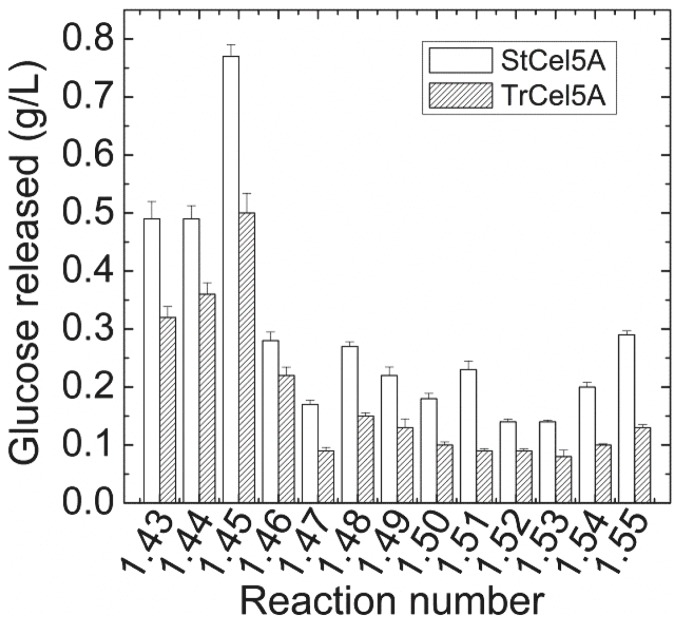
Comparison of StCel5A and TrCel5A on Glc yields from corn stover in selected reactions in the first optimization experiment. In each pair of reactions, the proportions of all of the enzymes except StCel5A and TrCel5A were identical. Table S1 shows the complete experimental results.

Results of the first optimization experiment are shown in Table S1. Only Glc release was assayed in this experiment. A subset of the results (i.e., reactions 1.43–1.55), in which all enzymes except StCel5A and TrCel5A were in the same proportions and concentrations, are shown in Fig. 4. Reactions containing StCel5A consistently gave higher Glc yields than those containing TrCel5A. The enhancement of Glc yield ranged from 27% to 156%. These results confirm that StCel5A is responsible for at least part of the synergistic activity of the *S. thermophile* secretome.

### Second optimization experiment

The first optimization experiment suggested that StCel5A was substituting for *T. reesei* endoglucanases and/or xylanases. To test this hypothesis, a second experiment was performed with varying proportions of CBH1, EG1, EX2, EX3, and either TrCel5A or StCel5A (Table S2). The proportions of these five enzymes were allowed to vary between 5% and 60%. The proportions of CBH2, BX, BG and AA9 were fixed at 5% each. In this way, a 9-component mixture was reduced to a 5-component mixture and consequently the number of reactions needed to evaluate an augmented quadratic surface response model was reduced from 55 to 26.

**Figure 5:**
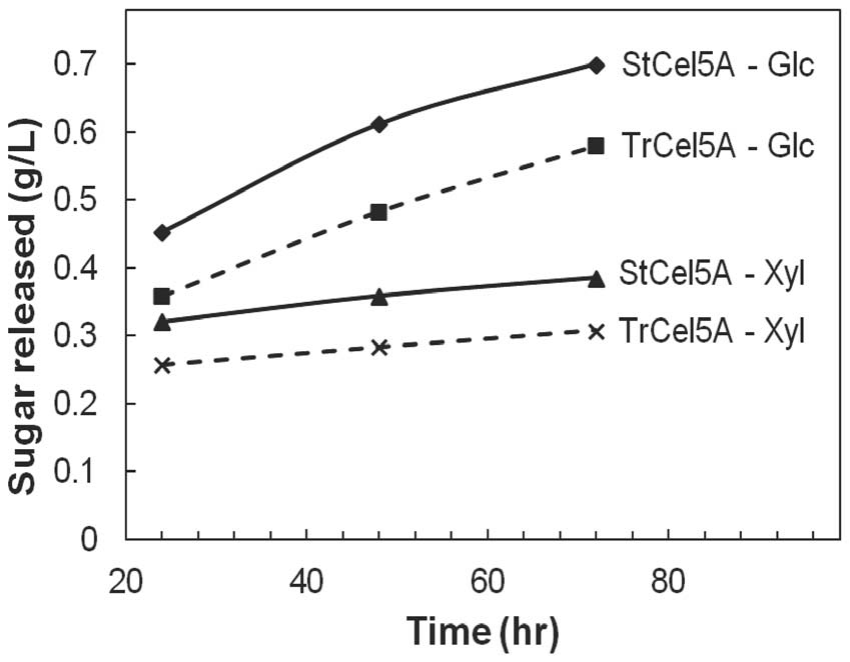
Yields of Glc and Xyl as a function of time and source of Cel5A for 8-CSM plus either StCel5A or TrCel5A. The experimental data are shown in Tables S3 and S4. Error bars (standard deviations) are smaller than the data symbols.

Both Glc and Xyl yields were measured after 24, 48, or 72 h (Tables S3 and S4). At the best enzyme mixture, StCel5A yielded more Glc and Xyl than TrCel5A (Fig. 5). The enhancement of Glc yields by StCel5A ranged from 9% to 15%, and the enhancement of Xyl yields ranged from 9% to10%.

**Figure 6:**
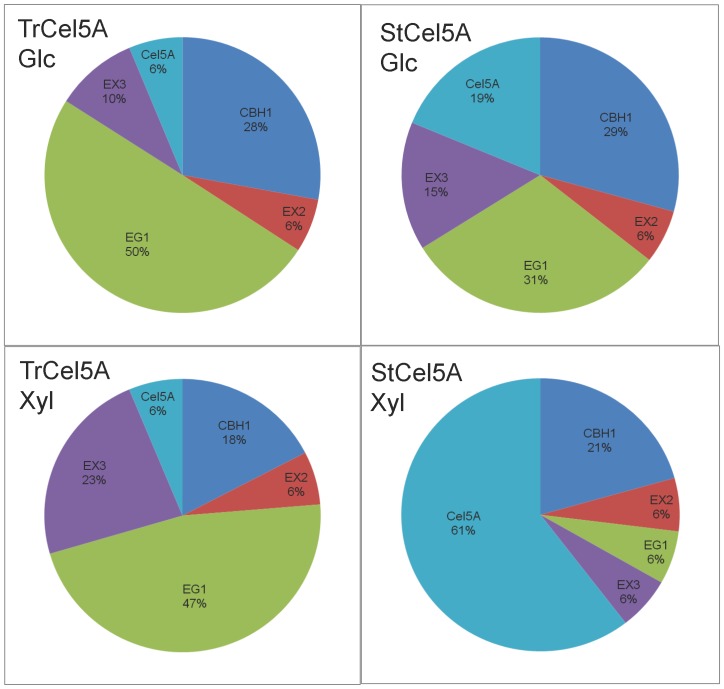
Optimal enzyme proportions for release of Glc or Xyl from corn stover after 72 h digestion with either TrCel5A or StCel5A. The other enzymes in the experiment (AA9, CBH2, BG, and BX) were fixed at 5% each. Glc and Xyl yields were 58% and 31% with TrCel5A and 70% and 39% with StCel5A, respectively. The data and statistical analyses are shown in Tables S3–S5.

The model predictions of optimized proportions of component enzymes after 72 h, based on the data in Tables S3 and S4, are shown in Fig. 6. For Glc, the optimal proportions for mixtures containing either TrCel5A or StCel5A were fairly similar for EX2, EX3, and CBH1. The largest difference was in the optimal proportions of EG1 (50% for TrCel5A vs. only 31% for StCel5A), and in the proportions of Cel5A itself (6% for TrCel5A and 19% for StCel5A). This reciprocal relationship between Cel5A and EG1 is consistent with both of them being endo-β1,4-glucanases.

For optimal Xyl release, however, the enzyme proportions showed much greater differences depending on whether TrCel5A or StCel5A was in the mixture (Fig. 6). Whereas the optimal proportions of CBH1 and EX2 did not change appreciably, switching from TrCel5A to StCel5A decreased the optimal level of EX3 from 23% to 6%, while Cel5A increased from 6% to 61%, and EG1 decreased from 47% to 6%. This result suggests a particularly complex interaction between xylanases and glucanases for the efficient release of Xyl, with marked differences in behavior between TrCel5A and StCel5A.

### Comparisons of StCel5A and TrCel5A

The physical properties of StCel5A and TrCel5A are similar, with predicted M_r_'s of 42,385 and 44,227, pI's of 5.15 and 4.85, three and one N-glycosylation sites, and signal peptides of 16 and 21 amino acids, respectively. Therefore, none of these properties are likely to contribute to their different abilities to digest biomass.

#### pH and temperature

**Figure 7:**
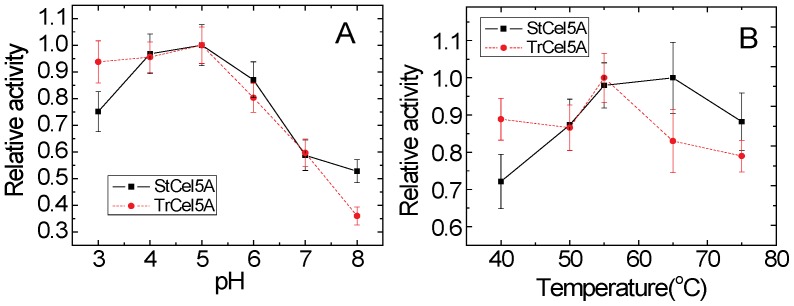
Hydrolytic activities of StCel5A or TrCel5A with lichenan as a function of (A) pH or (B) temperature.

The two enzymes showed similar pH profiles (Fig. 7A). Although StCel5A was earlier shown to have greater thermostability [14], our results indicated that the two enzymes were equally active at 50°C (Fig. 7B). Furthermore, when both enzymes were preincubated at 50°C for 48 hr and then assayed on lichenan for 48 hr, neither enzyme showed any detectable loss of activity (data not shown). Thus, neither pH nor temperature response appear to account for the differential effectiveness of TrCel5A and StCel5A.

#### Kinetic parameters

Tambor et al. [14] determined that the specificity constants (k_cat_/K_m_) for TrCel5A and StCel5A were 1.8 and 1.5 mL s^−1^ mg^−1^ on PASC and 6.1 and 15 mL s^−1^ mg^−1^ on CM-cellulose. In a standardized side-by-side assay, 1.8 pmol of either TrCel5a or StCel5A produced 0.17 and 0.22 nmol reducing sugars, respectively, from Avicel. Hydrolysis of 2% PASC in 20 h required 1.43 pmol of TrCel5A and 2.05 pmol of StCel5A [14]. Although not conclusive, these values do not seem sufficiently different to account for the superiority of StCel5A at releasing Glc from corn stover when combined with other glycosyl hydrolases, especially considering the intrinsic uncertainty in kinetic measurements. Neither enzyme was tested on lichenan, xylan, or mannan in the prior study [14].

#### Substrate specificities

GH5 is a large family of glycosyl hydrolases, and members have a range of activities, including β1,4-glucanase, lichenanase, β1,4-xylanase, xyloglucanase, β1,4-mannanase, β1,6-galactanase, β1,3-glucosidase , and β-fucosidase [26]. To determine if the superiority of StCel5A might be related to substrate specificity, it was compared against TrCel5A on an assortment of polysaccharide substrates.

**Figure 8:**
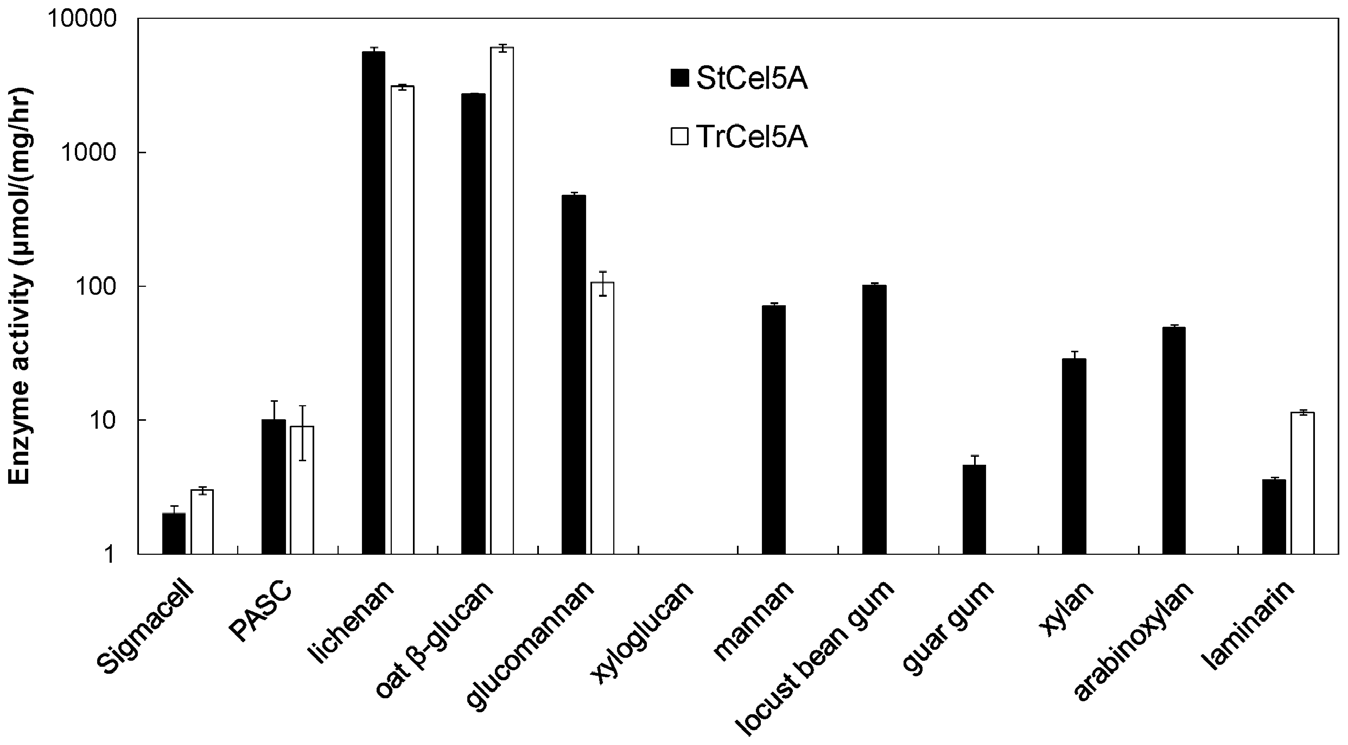
Activities of pure TrCel5A and StCel5A on defined substrates. Sigmacell is crystalline cellulose; PASC is phosphoric-acid swollen cellulose; lichenan and β-glucan are two types of mixed-linkage β1,3-β1,4 glucan, one from Iceland moss and the other from oats; xyloglucan is β1,4-linked glucan with α1,6-linked Xyl side chains from tamarind; mannan is insoluble β1,4-mannan; locust bean gum and guar gum are two types of β1,4-linked mannan with side chains of α1,6-linked galactose; xylan is β1,4-linked xylan; arabinoxylan is β1,4-linked xylan with α1,6-linked arabinose side chains; laminarin is predominantly β1,3-linked glucan. Shaded bars, StCel5A; open bars, TrCel5A. TrCel5A had no detectable activity against mannan, either galactomannan, xylan, or arabinoxylan. Neither enzyme had detectable activity against xyloglucan.

Both enzymes were active on lichenan and β-glucan (mixed-linked glucan), and glucomannan, and slightly active on laminarin (β1,3-glucan), crystalline cellullose (Sigmacell), and PASC (Fig. 8). Note that “glucomannan” is a mainly unbranched polymer with alternating β1,4-linked Glc and Man within the backbone, whereas “galactomannan” has a β1,4-linked mannan backbone with α-linked Gal side chains. StCel5A but not TrCel5A showed activity on the two galactomannans (locust bean gum and guar gum) and on the two xylans, one of which is substituted (arabinoxylan) and one which is not (xylan) (Fig. 8). Neither enzyme was active on xyloglucan. Therefore, StCel5A and TrCel5A differ qualitatively in their substrate specificities, and this might be a clue to their differential effectiveness at releasing Glc and Xyl from a complex biomass substrate in combination with other cell-wall active enzymes (Figs. 4–6).

We attempted to measure StCe5A-catalyzed release of Man from corn stover in an optimized 9-component enzyme cocktail. No Man could be detected (data not shown).

### Phylogenetic analysis of StCel5A, TrCel5A, and related GH5 cellulases

To explore further the reasons for superior performance of StCel5A and the possible involvement of substrate specificity, additional fungal members of GH5 were analyzed. By the classification of Aspeborg et al. [27], StCel5A and TrCel5A are both in subfamily 5 of GH5, denoted herein as GH5_5. Both have canonical CBM1 modules and associated linker regions following the signal peptides at the N termini. The full-length proteins share 35.3% amino acid identity and the catalytic domains share 32.3% identity. In the tree of GH5_5, there are 17 nodes separating StCel5A and TrCel5A [27]. All of the intervening proteins are of fungal origin with the exception of a single plant protein (BAK01092), which is probably an artifact. All of these proteins are known or predicted to belong to EC 3.2.1.4.

A phylogenenetic tree of just the fungal sequences in GH5_5 was constructed. Within GH5_5, CBM's are common but not universal, and some have C-terminal CBM's. Additional fungal members of GH5_5 that were present in the JGI database but not CAZy or GenBank (as of August, 2013), and therefore not in Aspeborg et al. [27], were added. On the other hand, not all of the sequences between StCel5A and TrCel5A in the GH5_5 tree nor all of the additional high scoring sequences in GenBank and JGI were included. Duplicates (i.e., sharing >95% amino acid identity) were excluded, and only proteins with N-terminal CBM's, like StCel5A and TrCel5A themselves, were included to remove this feature as a potentially confounding variable. The exception to this exclusion rule was TaCel5A from *Thermoascus aurantiacus*, which lacks a native CBM but was nonetheless included because it has a solved crystal structure (GenBank accession AAL88714, PDB ID 1GZJ) [28], [29].

**Figure 9:**
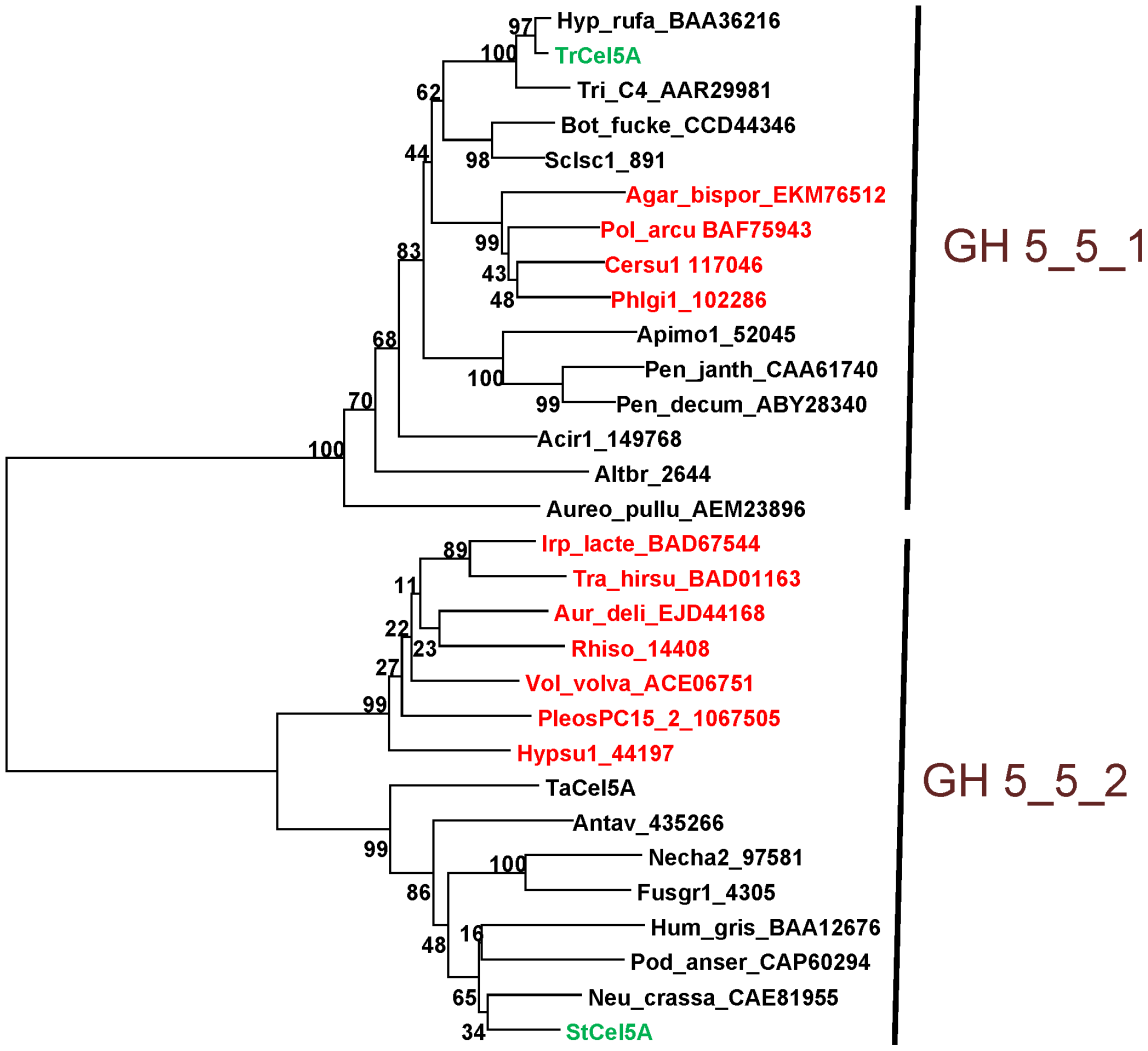
Alignment of the catalytic domains of fungal proteins in subfamily 5 of GH5 (GH5_5) showing its further division into two subclades, GH5_5_1 and GH5_5_2. All of the sequences have N-terminal CBM's except TaCel5A. StCel5A and TrCel5A are show in green. Sequences from Basidiomycota are shown in red; all other sequences are from Ascomycota. Species abbreviations and taxonomic affiliations are shown in Table S6.

Before alignment, the signal peptides, CBM's, and linkers (amounting to ∼95 amino acids) were removed, on the assumption that the basis of enzymatic superiority resides in the catalytic domain. The alignment of the resulting protein set, composed of 30 proteins of broad taxonomic distribution within the higher fungi, revealed two subfamilies within GH5_5, which we herein designate GH5_5_1 and GH5_5_2 (Fig. 9). TrCel5A falls into family GH5_5_1 whereas StCel5A falls into subfamily GH5_5_2 (Fig. 9). The separation of the fungal members of GH5_5 into two clades is also apparent in the phylogenetic tree of Aspeborg et al. [27], and all of the sequences in common between the two trees are congruent in regard to their membership in the two GH5_5 subfamilies. Examples from both Ascomycota and Basidiomycota are present in both GH5_5_1 and GH5_5_2, which suggests that the resolution into these two subfamilies reflects a functional specialization that predates the split between these two phyla, and not merely the taxonomic relatedness of the source organisms.

In regard to whether GH5_5 subfamily is correlated with enzymatic superiority and/or substrate specificity, biochemical information is not available for most of the proteins in GH5_5. However, TaCel5A, which shares 65% amino acid identity with StCel5A and 34% identity with TrCel5A, does not use xylan as a substrate, unlike StCel5A [30], [31]. EG1 of *Volvariella volvacea* (accession ACE06751), which is also in GH5_5_2, is also inactive on xylan [32]. Based on these two counter-examples, the trait of substrate specificity does not strictly correlate with the subfamilies shown in Fig. 9. It therefore remains to be determined whether the trait of superiority, as defined by our particular assays, correlates with subfamily within GH5_5.

### Comparisons of the known and deduced structures of TrCel5A, StCel5A, and TaCel5A

Conservation of amino acids were compared between StCel5A, TrCel5A, and other members of GH5_5. The following amino acids were 100% conserved in all of the sequences (numbering is based on the Met start of TrCel5A): Glu259 and Glu148 (active site nucleophile and general base, respectively), Arg60, His104, Asn105, Phe124, Asn147, His218, Tyr220, and Trp292. These are all known or thought to be important for catalysis and/or substrate binding in GH5 [28], [29], [33], [34].

**Figure 10:**
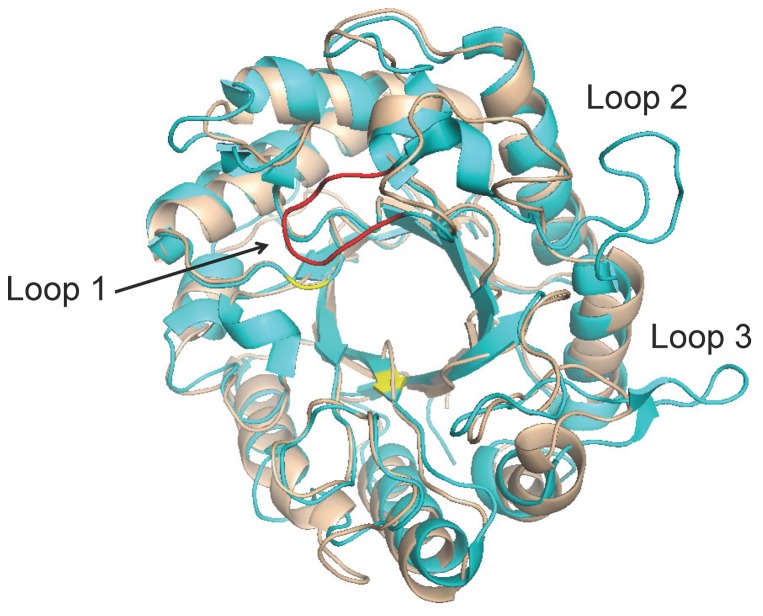
Modeled structure of StCel5A (grey) based on the structure of TaCel5A (PDB ID: 1GZJ) superimposed on TrCel5A (PDB ID: 3QR3, shown in blue). The two active-site Glu residues are shown in yellow. Loops 1, 2, and 3 are present in TrCel5A but not StCel5A; loop1 is shown in red.

The tertiary structure of StCel5A was modeled on the known structure of TrCel5A (Fig. 10). When superimposed on TrCel5A, the RMSD of the model calculated using the Align function in PyMol was 0.950 Å. TrCel5A has three loops that are predicted to be absent from StCel5A. Whereas loops 2 and 3 are far from the active site, loop 1 is predicted to be near the active site cleft (Fig. 10). This loop is also missing in TaCel5A (PDB ID: 1GZJ). The absence of this loop in both TaCel5A and StCel5A is consistent with their classification in subfamily 2 of GH5_5 (Fig. 9). However, the two Cys residues that form a disulfide bond in loop 1 of TrCel5A are present in some but not all members of the GH5_5_1 family.

**Figure 11:**
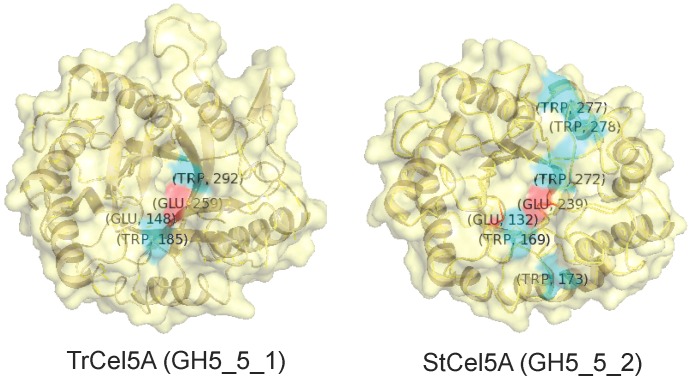
Surface views of StCel5A and TrCel5A showing Trp residues (blue) that are conserved in all members of GH5_5_1 (e.g., TrCel5A) or GH5_5_2 (e.g., StCel5A). The active-site Glu residues are shown in red. For orientation, the loop at the top of TrCel5A is the loop 2 shown in Fig. 10.

Potentially significant amino acids that are conserved within a subfamily, but not between subfamilies, include Gly189, which is always Trp in family 2 but Gly, Ala, or Glu in family 1; Gly291, which is always Trp in family 2 but Gly or Ser in family 1; Phe297, which is always Trp in family 2 and always Phe in family 1; and Asp298, which is always Trp in family 2 but never in family 1 (the most common substitutions are Asp or Gln). That is, four Trp residues are conserved in GH5_5_2 but not GH5_5_1. This might be significant in regard to the different behaviors (superiority and substrate range) of StCel5A and TrCel5A because Trp residues are involved in substrate binding through ring stacking in many glycosyl hydrolases [35]. For example, Trp170, Trp174, Trp273, Trp 278, and Trp279 line the binding cleft of TaCel5A [28], [29]. The corresponding amino acids in StCel5A are Trp169, Trp173, Trp272, Trp277, and Trp278, and in TrCel5A the corresponding amino acids are Trp185, Gly189, Trp292, Phe297, and Asp298. The conserved and subfamily-unique Trp residues in the binding cleft of GH5_5_1 and GH 5_5_2 are illustrated in Fig. 11.

## Discussion

In this paper, we report the re-identification of an endo-β1,4-glucanase from *S. thermophile* based on its synergistic activity at releasing fermentable sugars when tested in combination with a core set of eight enzymes from *T. reesei* on a realistic biomass substrate. In side-by-side comparisons, StCel5A was consistently superior at releasing both Glc and Xyl. Optimization experiments suggested that superiority was related to xylanase activity, and StCel5A, but not TrCel5A, was shown to utilize xylan as a substrate.

There are many previous reports of synergism between the cell-wall active enzymes of different micro-organisms [7], [11], [36], and this type of synergism can have multiple explanations. For example, some micro-organisms make enzymes that others do not [37]–[40], and enzymes from different sources with nominally the same catalytic activity can have different and superior pH optima, thermal stability, or resistance to denaturation.

The current work suggests that altered substrate specificity might be an additional contributor to synergistic activity. The involvement of broader substrate specificity in the superiority of StCel5A over TrCel5A is supported by the optimization experiments, in which the optimal proportions of the nine component enzymes was dependent on whether TrCel5A or StCel5A were included in the mixtures. The difference in optimal proportions was especially pronounced for yields of Xyl compared to Glc. All of our enzyme mixtures contained endo-β1,4-xylanases of GH10 (EX3) and GH11 (EX2), but xylanases in GH5 are known to have a different, generally broader, substrate range than either of these canonical xylanase families. Notably, GH5 xylanases have the ability to hydrolyze xylans that are substituted with glucuronic acid [26]. Therefore, StCel5A, which has xylanase activity in addition to β1,4-glucanase activity, may be superior to TrCel5A for Xyl release because it can degrade xylans that are not substrates for either EX2 or EX3 [41]–[43]. The xylanase activity of StCel5A might also explain why StCel5A also promotes Glc yields, because xylanases are known to enhance the accessibility of cellulose to cellulases [11]. StCel5A also had better activity than TrCel5A against various β1,4-mannans, including two types of galactomannan and unsubstituted mannan (Fig. 8). Because corn stover contains small amounts of galactose or Man compared to Glc and Xyl [3], it is unlikely that mannanase activity instead of xylanase activity accounts for the superiority of StCel5A, and, in fact, no release of Man from corn stover could be measured with StCel5A. Furthermore, we earlier showed in a 16-component optimization experiment that β-mannanase makes no contribution to Glc or Xyl release from pretreated corn stover (although it was one of the most important enzymes for release of Glc from dried distillers' grains) [3].

A structural understanding of the basis of substrate specificity in glycosyl hydrolases has been challenging. Chen et al. [44] analyzed the basis of substrate discrimination between glucan and galactomannan across the entire GH5 family. They identified a motif of six amino acids that when mutated altered substrate specificity, using Cel5A from the bacterium *Thermotaga maritima* (TmCel5A, AAD36816) as the model. TmCel5A is in subfamily 25 of GH5 [27] and can hydrolyze both cellulose and galactomannan, like StCel5A. Four of the six critical amino acids identified by Chen et al. [44] could be unambiguosly identified in StCel5A and TrCel5A despite low overall (<20%) amino acid identity. Among these six critical amino acids, His95 (TmCel5A numbering) was invariant in all three sequences, while the other residues varied but in a pattern that did not correlate with the known substrate specificity of the three enzymes. For example, in TmCel5A, TrCel5A, or StCel5A, respectively, amino acid 53 was Pro, Pro, or Asp; amino acid 96 was His, Asn, or Asn; and amino acid 287 was Asp, Ala, or Gly. Thus, substrate discrimination appears to follow different rules in subfamily 5 of GH5 than in subfamily 25.

The superiority of StCel5A over TrCel5A might also be due to features of the two enzymes that are unrelated to substrate specificity. This is supported by the fact that two other members of GH5_5_2 are not active against xylan, unlike StCel5A. Another consideration is that the critical feature(s) of StCel5A that cause superiority might manifest themselves only in combination with other necessary biomass-degrading enzymes (i.e., the 8-component synthetic mixture) and a real biomass substrate (i.e., pretreated corn stover). Therefore, the critical differences might not be observable in any experiments using pure individual enzymes and pure substrates, including the enzyme kinetic [14] and substrate specificity experiments (Fig. 8). Potentially critical properties of an enzyme that might be apparent in experiments involving enzyme mixtures and real biomass but not in isolation on pure substrates include nonproductive binding to one or more biomass components such as lignin, enhanced resistance to inhibitors in biomass, or inhibition by the products of other enzymes.

Further studies, including biochemical analysis of additional members of GH5_5, should resolve the structural features that are responsible for superiority, substrate specificity, and phylogenetic relatedness within this subfamily.

## Supporting Information

Table S1 **Design and results of first optimization experiment.**
(DOCX)

Table S2 **Design and results of first optimization experiment.**
(DOCX)

Table S3 **Glc yields (g/L) in the second optimization experiment based on the experimental design shown in Table S2.**
(DOCX)

Table S4 **Xyl yields (g/L) from the second optimization experiment.**
(DOCX)

Table S5 **Statistical analyses of the optimization experiments shown in Tables S3 and S4 as generated by DesignExpert.**
(DOCX)

Table S6 **Species abbreviations and taxonomic affiliations for the fungi shown in** **Fig. 9** **.**
(DOCX)
