# Supplementary material for: Enhancement of Synthetic Trichoderma-Based Enzyme Mixtures for Biomass Conversion with an Alternative Family 5 Glycosyl Hydrolase from Sporotrichum thermophile
Source: PLoS One. 2014 Oct 8;9(10):e109885. doi: 10.1371/journal.pone.0109885 (PMC4190410; doi:10.1371/journal.pone.0109885)
Supplement: Table S1 — Design and results of first optimization experiment. (DOCX) [file pone.0109885.s001.docx]

**Supplementary Table S1.** Design and results of first optimization experiment. Enzyme composition is expressed as a mass % of the total loading, which was fixed at 15 mg/gm corn stover glucan in every reaction. Only reactions 1.43-1.55 contained either StCel5A or TrCel5A (shown in Fig. 4). For these reactions, the upper value is the Glc yield obtained with StCel5A and the lower value is the yield obtained with TrCel5A. Values are expressed as the mean of 8 replicates ± 1 standard deviation of the mean (SD).

| Reaction number | CBH1 | CBH2 | EG1 | EX2 | EX3 | BX | BG | AA9 | **StCel5A or TrCel5A** | Glc Yield |
| --- | --- | --- | --- | --- | --- | --- | --- | --- | --- | --- |
| 1.1 | 0.05 | 0.22 | 0.05 | 0 | 0.18 | 0.2 | 0.26 | 0.04 | 0 | 0.21 ± 0.02 |
| 1.2 | 0.47 | 0.43 | 0.05 | 0 | 0 | 0 | 0.05 | 0 | 0 | 0.15 ± 0.01 |
| 1.3 | 0.3 | 0 | 0.05 | 0 | 0 | 0 | 0.65 | 0 | 0 | 0.06 ± 0.00 |
| 1.4 | 0.05 | 0.42 | 0.05 | 0 | 0 | 0.43 | 0.05 | 0 | 0 | 0.10 ± 0.01 |
| 1.5 | 0.05 | 0 | 0.05 | 0 | 0.65 | 0.2 | 0.05 | 0 | 0 | 0.05 ± 0.00 |
| 1.6 | 0.05 | 0.25 | 0.65 | 0 | 0 | 0 | 0.05 | 0 | 0 | 0.18 ± 0.00 |
| 1.7 | 0.05 | 0.65 | 0.22 | 0.03 | 0 | 0 | 0.05 | 0 | 0 | 0.23 ± 0.04 |
| 1.8 | 0.65 | 0 | 0.05 | 0.25 | 0 | 0 | 0.05 | 0 | 0 | 0.14 ± 0.01 |
| 1.9 | 0.05 | 0 | 0.65 | 0.25 | 0 | 0 | 0.05 | 0 | 0 | 0.07 ± 0.00 |
| 1.10 | 0.05 | 0.39 | 0.05 | 0.46 | 0 | 0 | 0.05 | 0 | 0 | 0.17 ± 0.01 |
| 1.11 | 0.05 | 0 | 0.25 | 0.65 | 0 | 0 | 0.05 | 0 | 0 | 0.06 ± 0.00 |
| 1.12 | 0.48 | 0 | 0.47 | 0 | 0 | 0 | 0.05 | 0 | 0 | 0.14 ± 0.01 |
| 1.13 | 0.05 | 0 | 0.48 | 0 | 0.42 | 0 | 0.05 | 0 | 0 | 0.07 ± 0.00 |
| 1.14 | 0.05 | 0 | 0.05 | 0.43 | 0.42 | 0 | 0.05 | 0 | 0 | 0.05 ± 0.00 |
| 1.15 | 0.47 | 0 | 0.05 | 0 | 0.43 | 0 | 0.05 | 0 | 0 | 0.15 ± 0.01 |
| 1.16 | 0.05 | 0.44 | 0.05 | 0 | 0.41 | 0 | 0.05 | 0 | 0 | 0.20 ± 0.02 |
| 1.17 | 0.05 | 0 | 0.05 | 0.42 | 0 | 0.43 | 0.05 | 0 | 0 | 0.05 ± 0.02 |
| 1.18 | 0.47 | 0 | 0.05 | 0 | 0 | 0.43 | 0.05 | 0 | 0 | 0.06 ± 0.02 |
| 1.19 | 0.05 | 0 | 0.46 | 0 | 0 | 0.44 | 0.05 | 0 | 0 | 0.05 ± 0.00 |
| 1.20 | 0.05 | 0 | 0.05 | 0 | 0.2 | 0.65 | 0.05 | 0 | 0 | 0.05 ± 0.00 |
| 1.21 | 0.05 | 0 | 0.05 | 0 | 0.2 | 0.65 | 0.05 | 0 | 0 | 0.05 ± 0.00 |
| 1.22 | 0.29 | 0 | 0.05 | 0.37 | 0 | 0 | 0.29 | 0 | 0 | 0.10 ± 0.00 |
| 1.23 | 0.29 | 0 | 0.05 | 0.37 | 0 | 0 | 0.29 | 0 | 0 | 0.10 ± 0.01 |
| 1.24 | 0.65 | 0 | 0.05 | 0 | 0 | 0 | 0.3 | 0 | 0 | 0.07 ± 0.00 |
| 1.25 | 0.05 | 0 | 0.05 | 0 | 0.43 | 0 | 0.47 | 0 | 0 | 0.05 ± 0.00 |
| 1.26 | 0.05 | 0.42 | 0.05 | 0 | 0 | 0 | 0.48 | 0 | 0 | 0.11 ± 0.00 |
| 1.27 | 0.05 | 0 | 0.47 | 0 | 0 | 0 | 0.48 | 0 | 0 | 0.06 ± 0.00 |
| 1.28 | 0.05 | 0 | 0.05 | 0 | 0 | 0.42 | 0.48 | 0 | 0 | 0.04 ± 0.00 |
| 1.29 | 0.05 | 0 | 0.05 | 0.25 | 0 | 0 | 0.65 | 0 | 0 | 0.05 ± 0.00 |
| 1.30 | 0.05 | 0 | 0.05 | 0 | 0 | 0.65 | 0.05 | 0.2 | 0 | 0.04 ± 0.01 |
| 1.31 | 0.05 | 0 | 0.25 | 0.05 | 0.2 | 0 | 0.24 | 0.22 | 0 | 0.09 ± 0.00 |
| 1.32 | 0.05 | 0 | 0.05 | 0 | 0.27 | 0.33 | 0.05 | 0.25 | 0 | 0.06 ± 0.00 |
| 1.33 | 0.05 | 0 | 0.05 | 0 | 0.27 | 0.33 | 0.05 | 0.25 | 0 | 0.07 ± 0.00 |
| 1.34 | 0.05 | 0.44 | 0.05 | 0 | 0 | 0 | 0.05 | 0.41 | 0 | 0.15 ± 0.02 |
| 1.35 | 0.05 | 0 | 0.05 | 0 | 0.44 | 0 | 0.05 | 0.41 | 0 | 0.06 ± 0.00 |
| 1.36 | 0.48 | 0 | 0.05 | 0 | 0 | 0 | 0.05 | 0.42 | 0 | 0.09 ± 0.00 |
| 1.37 | 0.05 | 0 | 0.48 | 0 | 0 | 0 | 0.05 | 0.42 | 0 | 0.07 ± 0.01 |
| 1.38 | 0.05 | 0 | 0.05 | 0.42 | 0 | 0 | 0.05 | 0.43 | 0 | 0.06 ± 0.01 |
| 1.39 | 0.05 | 0 | 0.05 | 0 | 0 | 0 | 0.47 | 0.43 | 0 | 0.05 ± 0.00 |
| 1.40 | 0.05 | 0 | 0.05 | 0 | 0 | 0.2 | 0.05 | 0.65 | 0 | 0.05 ± 0.03 |
| 1.41 | 0.05 | 0 | 0.05 | 0 | 0 | 0.2 | 0.05 | 0.65 | 0 | 0.05 ± 0.01 |
| 1.42 | 0.54 | 0.14 | 0.18 | 0 | 0 | 0.04 | 0.05 | 0.06 | 0 | 0.25 ± 0.03 |
| 1.43 | 0.05 | 0.19 | 0.32 | 0.2 | 0 | 0 | 0.05 | 0 | 0.18 | 0.25 ± 0.02  0.16 ± 0.01 |
| 1.44 | 0.05 | 0.19 | 0.32 | 0.2 | 0 | 0 | 0.05 | 0 | 0.18 | 0.25 ± 0.01  0.18 0.01 |
| 1.45 | 0.28 | 0.24 | 0.05 | 0 | 0.05 | 0.01 | 0.05 | 0.1 | 0.24 | 0.39 ± 0.01  0.25 ± 0.02 |
| 1.46 | 0.06 | 0 | 0.24 | 0 | 0.19 | 0.19 | 0.07 | 0.03 | 0.24 | 0.14 ± 0.01 0.11 ± 0.01 |
| 1.47 | 0.05 | 0 | 0.65 | 0 | 0 | 0 | 0.05 | 0 | 0.25 | 0.09 ± 0.00  0.05 ± 0.00 |
| 1.48 | 0.05 | 0.49 | 0.05 | 0 | 0 | 0 | 0.05 | 0 | 0.36 | 0.14 ± 0.00 0.08 ± 0.02 |
| 1.49 | 0.05 | 0 | 0.05 | 0 | 0.43 | 0 | 0.05 | 0 | 0.42 | 0.11 ± 0.01  0.07 ± 0.01 |
| 1.50 | 0.05 | 0 | 0.05 | 0 | 0 | 0 | 0.05 | 0.42 | 0.43 | 0.09 ± 0.00  0.05 ± 0.00 |
| 1.51 | 0.47 | 0 | 0.05 | 0 | 0 | 0 | 0.05 | 0 | 0.43 | 0.12 ± 0.01  0.05 ± 0.00 |
| 1.52 | 0.05 | 0 | 0.05 | 0 | 0 | 0 | 0.47 | 0 | 0.43 | 0.07 ± 0.00  0.05 ± 0.00 |
| 1.53 | 0.05 | 0 | 0.05 | 0 | 0 | 0.42 | 0.05 | 0 | 0.43 | 0.07 ± 0.00  0.04 ± 0.01 |
| 1.54 | 0.05 | 0 | 0.05 | 0.41 | 0 | 0 | 0.05 | 0 | 0.44 | 0.10 ± 0.00  0.05 ± 0.00 |
| 1.55 | 0.05 | 0.08 | 0.22 | 0 | 0 | 0 | 0.05 | 0 | 0.6 | 0.15 ± 0.00  0.07 ± 0.00 |
