# Supplementary material for: Enhancement of Synthetic Trichoderma-Based Enzyme Mixtures for Biomass Conversion with an Alternative Family 5 Glycosyl Hydrolase from Sporotrichum thermophile
Source: PLoS One. 2014 Oct 8;9(10):e109885. doi: 10.1371/journal.pone.0109885 (PMC4190410; doi:10.1371/journal.pone.0109885)
Supplement: Table S2 — Design and results of first optimization experiment. (DOCX) [file pone.0109885.s002.docx]

**Supplementary Table S2.** Experimental design for the second optimization experiment. Cel5A represents either TrCel5A or StCel5A. Numbers are the fractional content on a mass basis (total of each reaction was 15 mg/g corn stover glucan). Experimental results are shown in Tables S3 and S4. AA9 is also known as GH61.

| Reaction number | CBH1 | EX2 | EG1 | EX3 | **Cel5A** | BX | BG | AA9 | CBH2 |
| --- | --- | --- | --- | --- | --- | --- | --- | --- | --- |
| 2.1 | 0.6 | 0.05 | 0.05 | 0.05 | 0.05 | 0.05 | 0.05 | 0.05 | 0.05 |
| 2.2 | 0.05 | 0.6 | 0.05 | 0.05 | 0.05 | 0.05 | 0.05 | 0.05 | 0.05 |
| 2.3 | 0.05 | 0.05 | 0.6 | 0.05 | 0.05 | 0.05 | 0.05 | 0.05 | 0.05 |
| 2.4 | 0.05 | 0.05 | 0.05 | 0.6 | 0.05 | 0.05 | 0.05 | 0.05 | 0.05 |
| 2.5 | 0.05 | 0.05 | 0.05 | 0.05 | 0.6 | 0.05 | 0.05 | 0.05 | 0.05 |
| 2.6 | 0.32 | 0.32 | 0.05 | 0.05 | 0.05 | 0.05 | 0.05 | 0.05 | 0.05 |
| 2.7 | 0.32 | 0.05 | 0.32 | 0.05 | 0.05 | 0.05 | 0.05 | 0.05 | 0.05 |
| 2.8 | 0.32 | 0.05 | 0.05 | 0.32 | 0.05 | 0.05 | 0.05 | 0.05 | 0.05 |
| 2.9 | 0.32 | 0.05 | 0.05 | 0.05 | 0.32 | 0.05 | 0.05 | 0.05 | 0.05 |
| 2.10 | 0.05 | 0.32 | 0.32 | 0.05 | 0.05 | 0.05 | 0.05 | 0.05 | 0.05 |
| 2.11 | 0.05 | 0.32 | 0.05 | 0.32 | 0.05 | 0.05 | 0.05 | 0.05 | 0.05 |
| 2.12 | 0.05 | 0.32 | 0.05 | 0.05 | 0.32 | 0.05 | 0.05 | 0.05 | 0.05 |
| 2.13 | 0.05 | 0.05 | 0.32 | 0.32 | 0.05 | 0.05 | 0.05 | 0.05 | 0.05 |
| 2.14 | 0.05 | 0.05 | 0.32 | 0.05 | 0.32 | 0.05 | 0.05 | 0.05 | 0.05 |
| 2.15 | 0.05 | 0.05 | 0.05 | 0.32 | 0.32 | 0.05 | 0.05 | 0.05 | 0.05 |
| 2.16 | 0.38 | 0.1 | 0.1 | 0.1 | 0.1 | 0.05 | 0.05 | 0.05 | 0.05 |
| 2.17 | 0.1 | 0.38 | 0.1 | 0.1 | 0.1 | 0.05 | 0.05 | 0.05 | 0.05 |
| 2.18 | 0.1 | 0.1 | 0.38 | 0.1 | 0.1 | 0.05 | 0.05 | 0.05 | 0.05 |
| 2.19 | 0.1 | 0.1 | 0.1 | 0.38 | 0.1 | 0.05 | 0.05 | 0.05 | 0.05 |
| 2.20 | 0.1 | 0.1 | 0.1 | 0.1 | 0.38 | 0.05 | 0.05 | 0.05 | 0.05 |
| 2.21 | 0.16 | 0.16 | 0.16 | 0.16 | 0.16 | 0.05 | 0.05 | 0.05 | 0.05 |
| 2.22 | 0.6 | 0.05 | 0.05 | 0.05 | 0.05 | 0.05 | 0.05 | 0.05 | 0.05 |
| 2.23 | 0.05 | 0.6 | 0.05 | 0.05 | 0.05 | 0.05 | 0.05 | 0.05 | 0.05 |
| 2.24 | 0.05 | 0.05 | 0.6 | 0.05 | 0.05 | 0.05 | 0.05 | 0.05 | 0.05 |
| 2.25 | 0.05 | 0.05 | 0.05 | 0.6 | 0.05 | 0.05 | 0.05 | 0.05 | 0.05 |
| 2.26 | 0.05 | 0.05 | 0.05 | 0.05 | 0.6 | 0.05 | 0.05 | 0.05 | 0.05 |
