# Supplementary material for: Enhancement of Synthetic Trichoderma-Based Enzyme Mixtures for Biomass Conversion with an Alternative Family 5 Glycosyl Hydrolase from Sporotrichum thermophile
Source: PLoS One. 2014 Oct 8;9(10):e109885. doi: 10.1371/journal.pone.0109885 (PMC4190410; doi:10.1371/journal.pone.0109885)
Supplement: Table S4 — Xyl yields (g/L) from the second optimization experiment. (DOCX) [file pone.0109885.s004.docx]

**Supplementary Table S4.** Xyl yields (g/L) from the second optimization experiment. Experimental design is shown in Table S2. Reactions 27-52 are replicates of reactions 1-26. Each result is the mean of 4 replicates ± 1 SD.

|  | StCel5A (24 hr) | StCel5A (48 hr) | StCel5A (72 hr) | TrCel5A  (24 hr) | TrCel5A  (48 hr) | TrCel5A  (72 hr) |
| --- | --- | --- | --- | --- | --- | --- |
| 1 | 0.03 ± 0 | 0.04 ± 0 | 0.05 ± 0 | 0.02 ± 0.01 | 0.04 ± 0 | 0.04 ± 0 |
| 2 | 0.25 ± 0 | 0.29 ± 0 | 0.31 ± 0 | 0.23 ± 0.01 | 0.26 ± 0 | 0.27 ± 0 |
| 3 | 0.25 ± 0.01 | 0.30 ± 0 | 0.33 ± 0 | 0.21 ± 0.01 | 0.26 ± 0 | 0.28 ± 0 |
| 4 | 0.26 ± 0.02 | 0.29 ± 0 | 0.31 ± 0.01 | 0.24 ± 0 | 0.26 ± 0 | 0.28 ± 0 |
| 5 | 0.28 ± 0 | 0.33 ± 0 | 0.36 ± 0 | 0.2 ± 0.01 | 0.23 ± 0 | 0.26 ± 0 |
| 6 | 0.28 ± 0.01 | 0.29 ± 0 | 0.31 ± 0 | 0.25 ± 0 | 0.27 ± 0 | 0.29 ± 0 |
| 7 | 0.27 ± 0 | 0.32 ± 0 | 0.34 ± 0.01 | 0.25 ± 0.01 | 0.29 ± 0.01 | 0.30 ± 0 |
| 8 | 0.28 ± 0.01 | 0.31 ± 0 | 0.33 ± 0 | 0.25 ± 0 | 0.27 ± 0.01 | 0.29 ± 0 |
| 9 | 0.28 ± 0.01 | 0.33 ± 0 | 0.37 ± 0 | 0.22 ± 0 | 0.25 ± 0 | 0.27 ± 0 |
| 10 | 0.24 ± 0 | 0.29 ± 0.01 | 0.32 ± 0 | 0.24 ± 0.01 | 0.27 ± 0 | 0.29 ± 0 |
| 11 | 0.25 ± 0.02 | 0.28 ± 0 | 0.31 ± 0 | 0.26 ± 0.01 | 0.27 ± 0 | 0.29 ± 0 |
| 12 | 0.28 ± 0.01 | 0.32 ± 0.01 | 0.35 ± 0.01 | 0.22 ± 0.01 | 0.24 ± 0 | 0.26 ± 0 |
| 13 | 0.24 ± 0 | 0.32 ± 0 | 0.3 ± 0.01 | 0.24 ± 0.01 | 0.28 ± 0 | 0.31 ± 0 |
| 14 | 0.22 ± 0.02 | 0.34 ± 0 | 0.33 ± 0.01 | 0.23 ± 0 | 0.27 ± 0.01 | 0.29 ± 0.01 |
| 15 | 0.25 ± 0.01 | 0.36 ± 0.01 | 0.34 ± 0 | 0.24 ± 0.02 | 0.28 ± 0 | 0.30 ± 0 |
| 16 | 0.06 ± 0.02 | 0.06 ± 0 | 0.06 ± 0 | 0.04 ± 0 | 0.05 ± 0 | 0.05 ± 0 |
| 17 | 0.24 ± 0.01 | 0.33 ± 0 | 0.30 ± 0 | 0.23 ± 0.06 | 0.28 ± 0 | 0.30 ± 0.02 |
| 18 | 0.27 ± 0.02 | 0.33 ± 0 | 0.32 ± 0 | 0.23 ± 0.01 | 0.27 ± 0 | 0.30 ± 0 |
| 19 | 0.27 ± 0.01 | 0.32 ± 0 | 0.31 ± 0 | 0.25 ± 0.01 | 0.28 ± 0 | 0.30 ± 0 |
| 20 | 0.29 ± 0.03 | 0.35 ± 0 | 0.33 ± 0.01 | 0.24 ± 0.01 | 0.26 ± 0 | 0.29 ± 0 |
| 21 | 0.32 ± 0.01 | 0.37 ± 0 | 0.36 ± 0 | 0.26 ± 0.01 | 0.28 ± 0 | 0.30 ± 0.01 |
| 22 | 0.06 ± 0.04 | 0.05 ± 0 | 0.04 ± 0 | 0.04 ± 0.01 | 0.04 ± 0 | 0.04 ± 0 |
| 23 | 0.26 ± 0.01 | 0.28 ± 0 | 0.26 ± 0 | 0.22 ± 0.01 | 0.25 ± 0 | 0.26 ± 0 |
| 24 | 0.21 ± 0.03 | 0.29 ± 0 | 0.28 ± 0 | 0.22 ± 0 | 0.25 ± 0 | 0.28 ± 0 |
| 25 | 0.26 ± 0.02 | 0.31 ± 0 | 0.34 ± 0 | 0.25 ± 0.02 | 0.27 ± 0.01 | 0.29 ± 0 |
| 26 | 0.27 ± 0.01 | 0.33 ± 0 | 0.35 ± 0 | 0.2 ± 0.01 | 0.23 ± 0 | 0.25 ± 0 |
| 27 | 0.04 ± 0.01 | 0.05 ± 0 | 0.05 ± 0 | 0.02 ± 0.01 | 0.04 ± 0 | 0.04 ± 0 |
| 28 | 0.27 ± 0.02 | 0.29 ± 0 | 0.31 ± 0.01 | 0.22 ± 0.01 | 0.25 ± 0 | 0.27 ± 0 |
| 29 | 0.27 ± 0.02 | 0.29 ± 0 | 0.33 ± 0.01 | 0.22 ± 0 | 0.27 ± 0 | 0.30 ± 0.01 |
| 30 | 0.29 ± 0.01 | 0.31 ± 0 | 0.33 ± 0.01 | 0.26 ± 0.01 | 0.27 ± 0 | 0.30 ± 0.01 |
| 31 | 0.3 ± 0 | 0.32 ± 0 | 0.35 ± 0 | 0.2 ± 0.03 | 0.22 ± 0 | 0.25 ± 0 |
| 32 | 0.29 ± 0.01 | 0.31 ± 0 | 0.34 ± 0 | 0.25 ± 0.03 | 0.29 ± 0 | 0.31 ± 0 |
| 33 | 0.31 ± 0.01 | 0.32 ± 0.01 | 0.36 ± 0 | 0.23 ± 0.03 | 0.29 ± 0 | 0.31 ± 0 |
| 34 | 0.33 ± 0.01 | 0.34 ± 0 | 0.37 ± 0 | 0.25 ± 0.03 | 0.27 ± 0 | 0.30 ± 0.01 |
| 35 | 0.34 ± 0.01 | 0.36 ± 0 | 0.39 ± 0 | 0.22 ± 0.02 | 0.25 ± 0 | 0.27 ± 0 |
| 36 | 0.28 ± 0.01 | 0.32 ± 0 | 0.35 ± 0 | 0.23 ± 0.02 | 0.26 ± 0 | 0.28 ± 0 |
| 37 | 0.28 ± 0.01 | 0.31 ± 0 | 0.34 ± 0 | 0.24 ± 0 | 0.28 ± 0 | 0.30 ± 0 |
| 38 | 0.29 ± 0.03 | 0.33 ± 0 | 0.37 ± 0.02 | 0.22 ± 0.01 | 0.25 ± 0 | 0.26 ± 0 |
| 39 | 0.27 ± 0.01 | 0.33 ± 0 | 0.35 ± 0 | 0.25 ± 0.02 | 0.29 ± 0 | 0.31 ± 0 |
| 40 | 0.29 ± 0 | 0.33 ± 0 | 0.38 ± 0.01 | 0.21 ± 0.02 | 0.25 ± 0 | 0.27 ± 0.01 |
| 41 | 0.32 ± 0 | 0.35 ± 0 | 0.38 ± 0.01 | 0.24 ± 0.03 | 0.27 ± 0 | 0.29 ± 0 |
| 42 | 0.09 ± 0.01 | 0.06 ± 0 | 0.07 ± 0 | 0.04 ± 0 | 0.05 ± 0 | 0.05 ± 0 |
| 43 | 0.33 ± 0 | 0.32 ± 0 | 0.35 ± 0 | 0.25 ± 0.02 | 0.28 ± 0 | 0.30 ± 0 |
| 44 | 0.32 ± 0 | 0.34 ± 0 | 0.39 ± 0 | 0.24 ± 0.02 | 0.27 ± 0 | 0.30 ± 0.01 |
| 45 | 0.33 ± 0.02 | 0.34 ± 0 | 0.37 ± 0.01 | 0.26 ± 0.01 | 0.27 ± 0.01 | 0.30 ± 0 |
| 46 | 0.39 ± 0.01 | 0.35 ± 0.04 | 0.44 ± 0 | 0.21 ± 0.01 | 0.24 ± 0 | 0.27 ± 0 |
| 47 | 0.35 ± 0.02 | 0.37 ± 0 | 0.41 ± 0.01 | 0.25 ± 0.02 | 0.27 ± 0 | 0.30 ± 0.01 |
| 48 | 0.05 ± 0 | 0.04 ± 0 | 0.05 ± 0 | 0.03 ± 0.01 | 0.04 ± 0 | 0.05 ± 0 |
| 49 | 0.26 ± 0 | 0.30 ± 0 | 0.33 ± 0 | 0.24 ± 0 | 0.25 ± 0 | 0.27 ± 0 |
| 50 | 0.25 ± 0.01 | 0.29 ± 0 | 0.32 ± 0 | 0.22 ± 0.01 | 0.27 ± 0 | 0.29 ± 0.01 |
| 51 | 0.29 ± 0.01 | 0.31 ± 0 | 0.34 ± 0.01 | 0.24 ± 0 | 0.27 ± 0 | 0.29 ± 0 |
| 52 | 0.32 ± 0.01 | 0.34 ± 0 | 0.36 ± 0.01 | 0.18 ± 0.01 | 0.23 ± 0 | 0.25 ± 0 |
