# Supplementary material for: Enhancement of Synthetic Trichoderma-Based Enzyme Mixtures for Biomass Conversion with an Alternative Family 5 Glycosyl Hydrolase from Sporotrichum thermophile
Source: PLoS One. 2014 Oct 8;9(10):e109885. doi: 10.1371/journal.pone.0109885 (PMC4190410; doi:10.1371/journal.pone.0109885)
Supplement: Table S5 — Statistical analyses of the optimization experiments shown in Tables S3 and S4 as generated by DesignExpert. (DOCX) [file pone.0109885.s005.docx]

**Supplementary Table S5.** Statistical analyses of the optimization experiments shown in Tables S3 and S4 as generated by DesignExpert.

| Optimized response | p-value | F-value | R^2 | Adjusted R^2 | Predicted R^2 | Difference between Adj and Pred R^2 | Adequate Precision | Model  desirability |
| --- | --- | --- | --- | --- | --- | --- | --- | --- |
| StCel5A  24 hr Glc | <0.0001 | 9.27 | 0.78 | 0.70 | 0.67 | 0.03 | 11.20 | 0.892 |
| TrCel5A  24 hr Glc | <0.0001 | 5.81 | 0.69 | 0.57 | 0.51 | 0.06 | 9.44 | 0.881 |
| StCel5A  48 hr Glc | <0.0001 | 8.73 | 0.77 | 0.68 | 0.65 | 0.03 | 11.20 | 0.806 |
| TrCel5A  48 hr Glc | <0.0001 | 5.90 | 0.70 | 0.58 | 0.51 | 0.07 | 9.53 | 0.892 |
| StCel5A  72 hr Glc | <0.0001 | 7.92 | 0.75 | 0.66 | 0.62 | 0.04 | 10.31 | 0.876 |
| TrCel5A  72 hr Glc | <0.0001 | 5.99 | 0.70 | 0.58 | 0.52 | 0.06 | 9.58 | 0.897 |
| StCel5A  24 hr Xyl | <0.0001 | 7.82 | 0.75 | 0.66 | 0.61 | 0.05 | 12.09 | 0.732 |
| TrCel5A  24 hr Xyl | <0.0001 | 8.26 | 0.76 | 0.67 | 0.60 | 0.07 | 10.87 | 1 |
| StCel5A  48 hr Xyl | <0.0001 | 8.92 | 0.78 | 0.69 | 0.63 | 0.06 | 11.62 | 0.966 |
| TrCel5A  48 hrXyl | <0.0001 | 7.90 | 0.75 | 0.66 | 0.58 | 0.08 | 10.41 | 0.977 |
| StCel5A  72 hr Xyl | <0.0001 | 7.78 | 0.75 | 0.65 | 0.59 | 0.06 | 11.68 | 0.870 |
| TrCel5A  72 hr Xyl | <0.0001 | 7.92 | 0.75 | 0.66 | 0.58 | 0.08 | 10.45 | 0.983 |
