# Supplementary material for: Enhancement of Synthetic Trichoderma-Based Enzyme Mixtures for Biomass Conversion with an Alternative Family 5 Glycosyl Hydrolase from Sporotrichum thermophile
Source: PLoS One. 2014 Oct 8;9(10):e109885. doi: 10.1371/journal.pone.0109885 (PMC4190410; doi:10.1371/journal.pone.0109885)
Supplement: Table S6 — Species abbreviations and taxonomic affiliations for the fungi shown in Fig. 9 . (DOCX) [file pone.0109885.s006.docx]

**Supplementary Table S6.** Species abbreviations and taxonomic affiliations for the fungi shown in Fig. 9.

| **Abbreviation** | **Species** | **Taxonomic Classification** |
| --- | --- | --- |
| Hyp_rufa | *Hypocrea rufa* | Sordariomycetes |
| Tri_C-4 | *Trichoderma sp. C-4* | Sordariomycetes |
| Bot_fucke | *Botryotinia fuckeliana* | Leotiomycetes |
| Sclsc1 | *Sclerotinia sclerotiorum* | Leotiomycetes |
| Agar_bispor | *Agaricus bisporus* | Agaricomycotina |
| Pol_arcu | *Polyporus arcularius* | Agaricomycotina |
| Cersu1 | *Ceriporiopsis subvermispora* | Agaricomycotina |
| Phlgi1 | *Phlebiopsis gigantea* | Agaricomycotina |
| Apimo1 | *Apiospora montagnei* | Sordariomycetes |
| Pen_janth | *Penicillium janthinellum* | Eurotiomycetes |
| Pen_decum | *Penicillium decumbens* | Eurotiomycetes |
| Acir1 | *Acidomyces richmondensis* | Dothidiomycetes |
| Altbr | *Alternaria brassicicola* | Dothidiomycetes |
| Aureo_pullu | *Aureobasidium pullulans* | Dothidiomycetes |
| Irp_lacte | *Irpex lacteus* | Agaricomycotina |
| Tra_hirsu | *Trametes hirsutum* | Agaricomycotina |
| Aur_deli | *Auricularia delicata* | Agaricomycotina |
| Rhiso | *Rhizoctonia solani* | Agaricomycotina |
| Vol_volva | *Volvariella volvacea* | Agaricomycotina |
| PleosPC15_2 | *Pleurotus ostreatus* | Agaricomycotina |
| Hypsu1 | *Hypholoma sublateritium* | Agaricomycotina |
| Antav | *Anthostoma avocetta* | Sordariomycetes |
| Necha2 | *Nectria haematococca* | Sordariomycetes |
| Fusgr1 | *Fusarium graminearum* | Sordariomycetes |
| Hum_gris | *Humicola grisea* | Sordariomycetes |
| Pod_anser | *Podospora anserina* | Sordariomycetes |
| Neu_crassa | *Neurospora crassa* | Sordariomycetes |
